# Self-Care Behaviors of Ovarian Cancer Patients Before Their Diagnosis: Proof-of-Concept Study

**DOI:** 10.2196/10447

**Published:** 2019-01-17

**Authors:** James M Flanagan, Hanna Skrobanski, Xin Shi, Yasemin Hirst

**Affiliations:** 1 Division of Cancer Department of Surgery and Cancer Imperial College London London United Kingdom; 2 Institute of Epidemiology and Public Health Research Department of Behavioural Science and Health University College London London United Kingdom; 3 School of Health Sciences University of Surrey Guildford United Kingdom; 4 Business School Manchester Metropolitan University Manchester United Kingdom; 5 School of Management Shanghai University Shanghai China; 6 Karaganda State Medical University Karaganda Kazakhstan

**Keywords:** cancer, early diagnosis, proof of concept, focus group, acceptability, data linkage, cancer surveillance

## Abstract

**Background:**

Longer patient intervals can lead to more late-stage cancer diagnoses and higher mortality rates. Individuals may delay presenting to primary care with red flag symptoms and instead turn to the internet to seek information, purchase over-the-counter medication, and change their diet or exercise habits. With advancements in machine learning, there is the potential to explore this complex relationship between a patient’s symptom appraisal and their first consultation at primary care through linkage of existing datasets (eg, health, commercial, and online).

**Objective:**

Here, we aimed to explore feasibility and acceptability of symptom appraisal using commercial- and health-data linkages for cancer symptom surveillance.

**Methods:**

A proof-of-concept study was developed to assess the general public’s acceptability of commercial- and health-data linkages for cancer symptom surveillance using a qualitative focus group study. We also investigated self-care behaviors of ovarian cancer patients using high-street retailer data, pre- and postdiagnosis.

**Results:**

Using a high-street retailer’s data, 1118 purchases—from April 2013 to July 2017—by 11 ovarian cancer patients and one healthy individual were analyzed. There was a unique presence of purchases for pain and indigestion medication prior to cancer diagnosis, which could signal disease in a larger sample. Qualitative findings suggest that the public are willing to consent to commercial- and health-data linkages as long as their data are safeguarded and users of this data are transparent about their purposes.

**Conclusions:**

Cancer symptom surveillance using commercial data is feasible and was found to be acceptable. To test efficacy of cancer surveillance using commercial data, larger studies are needed with links to individual electronic health records.

## Introduction

Early diagnosis is key to increasing the chances of 10-year survival rates and the number of people living beyond cancer. However, when the symptoms first present, only a very small proportion of people believe that their symptoms might be a sign of cancer; failure to recognize the signs and symptoms of cancer have been strongly linked to delays in help-seeking [1,2]. While greater symptom awareness and body vigilance are a key part of the patient appraisal and help-seeking [3], it has been suggested that people might use past experiences to reassure themselves that their symptoms are normal [4]. Ovarian cancer symptoms (eg, feeling bloated and abdominal pain) [5,6] and women’s personal experiences are prime examples of how symptoms can be normalized and potentially lead to delays in diagnosis [7].

Epithelial ovarian cancer has no specific recognizable symptoms and, as a result, most women are diagnosed at a late stage when the cancer has already spread around the peritoneum and the prognosis is poor. Approximately 7400 new cases of ovarian cancer are diagnosed each year in the United Kingdom, with over 4000 women dying from the disease each year [8]. The 10-year survival rate is only 35% in the United Kingdom; the survival rate is dramatically different if patients are diagnosed earlier with stage 1 disease (90%) compared with stage 3 or 4 (5%-15%), which unfortunately includes the majority of patients. Given that screening with cancer antigen 125 (CA 125) and transvaginal ultrasound do not appear to reduce mortality associated with ovarian cancer [9,10], the key to reducing this mortality is earlier diagnosis among women who are symptomatic, identifying those at high risk, and prevention.

Women with ovarian cancer usually report to primary care with symptoms at least six months before diagnosis; this suggests that symptom presentation and management are key parts of understanding ovarian cancer prognosis better [11]. A previous study showed a third of patients with ovarian cancer receive prescription medication to manage irritable bowel disease, constipation, stress, and depression before being diagnosed with cancer [12]. Qualitative studies on patients’ symptoms appraisal support the fact that women with ovarian cancer self-medicate their symptoms before they become debilitating [7]. If ovarian cancer symptoms overlap with patients’ sense of self and normality before they are perceived as signs of pathology [13], persistent use of over-the-counter medication could be an indication of ovarian cancer.

Most self-care evidence prior to diagnosis comes from retrospective studies with cancer patients, by the use of self-reported data from surveys and qualitative interviews [14]. Although they are important in understanding what may have caused delays in help-seeking, they have limited applications in real-life interventions. On the contrary, *big data* refers to massive amounts of data collected at rapid and efficient rates due to technological advances [15]. Big data in health care has the major potential to connect information from different sources to generate real-time datasets and outputs to monitor illnesses [16,17]. For instance, recent studies have utilized digital data to gain a better understanding of online health-information-searching by conducting large-scale analyses of search engine logs. By analyzing the sequence of terms inputted about health, studies have demonstrated the ability to detect influenza [18] and dengue [19] outbreaks, to discover side effects of medications [20], to assess effectiveness of internet-based preventative health programs [21], and to predict the changing information needs of women with breast cancer, from diagnosis to treatment [22].

Furthermore, a recent study has shown the feasibility of using online search terms describing cancer-relevant symptoms to predict forthcoming diagnoses of early-stage pancreatic cancer [23]. In addition to the use of online search engine logs to forecast early signs of cancer, future studies could use other sources of commercial data (ie, loyalty card and tracker data, as well as social media data collected by commercial organizations to understand consumer behaviors) to further understand how people evaluate and implement self-care for their cancer symptoms. However, one of the key challenges of using personal, commercial big data in cancer research is not knowing whether using commercial data to predict cancer is an acceptable approach within this decade, and whether it will provide meaningful insights into symptom appraisal and help-seeking.

Here, we aimed to evaluate inquiries on both acceptability and feasibility of cancer symptom surveillance using commercial data with a proof-of-concept study. Proof-of-concept studies are used to establish whether the proposed methodology or the concept is valid and feasible [24]. We used ovarian cancer as our primary cancer for our case study. We investigated the proof-of-concept evidence within the purchasing behaviors of women pre- and postdiagnosis using data from a high-street retailer that contains purchases of pain and indigestion medications. Furthermore, to better understand public attitudes and whether this project can be carried out with prospective real-time data, we assessed the acceptability of commercial- and health-data linkage for cancer symptom surveillance among a healthy population.

## Methods

### Ovarian Cancer Case Study

#### Study Design and Setting

We conducted a retrospective study of purchasing behaviors using ovarian cancer patients’ pre- and postdiagnosis data held in connection with a high-street retailer loyalty card. The study was facilitated by the Economic and Social Research Council (ESRC)-funded Consumer Data Research Centre (CDRC), which is based at University College London (UCL), London, United Kingdom. The CDRC has a license agreement with the high-street retailer, which agreed to support the study. Under CDRC guidelines, the data we requested were considered controlled data, which are defined as “data which need to be held under the most secure conditions with stringent access restrictions.” This meant that all data analysis was performed at a secure data laboratory based at UCL. JMF and YH were the only people with access to the data.

#### Data Collection

With the support from a patient representative group from a charity, Ovarian Cancer Action, 70 patients who were not under treatment for ovarian cancer from January to May 2017 received an invitation pack, including a study information sheet, a self-report survey, a consent form, and a free-post envelope. Once consent forms and surveys were returned, the researcher provided the high-street retailer with the unique loyalty card ID and a unique study ID for each of the consenting participants. The high-street retailer extracted data to be transferred into the CDRC secure lab using an encrypted server. The researchers used the unique study IDs to merge the survey data with the retailer data. The individuals’ data collected through the survey were not accessible to the retailer and the CDRC. Due to time restrictions, we included one healthy subject in the study.

#### Self-Report Survey

A self-report survey was designed to obtain information about the timeline of the cancer diagnosis, symptoms observed, demographics, and the loyalty card usage (see Multimedia Appendix 1). We asked the participants to report the first time they recognized signs and symptoms, the first time they booked an appointment with a health care professional, and the month and year of diagnosis. The symptoms included irregular periods or vaginal bleeding after menopause, back pain, lower-tummy pain, passing urine more than usual, constipation, pain during sex, weight loss, persistent bloating, loss of appetite, and feeling tired. Patients were given *other* as a response option. In addition, the survey recorded self-reported purchases of over-the-counter medication.

#### Data Analysis

**Figure 1:**
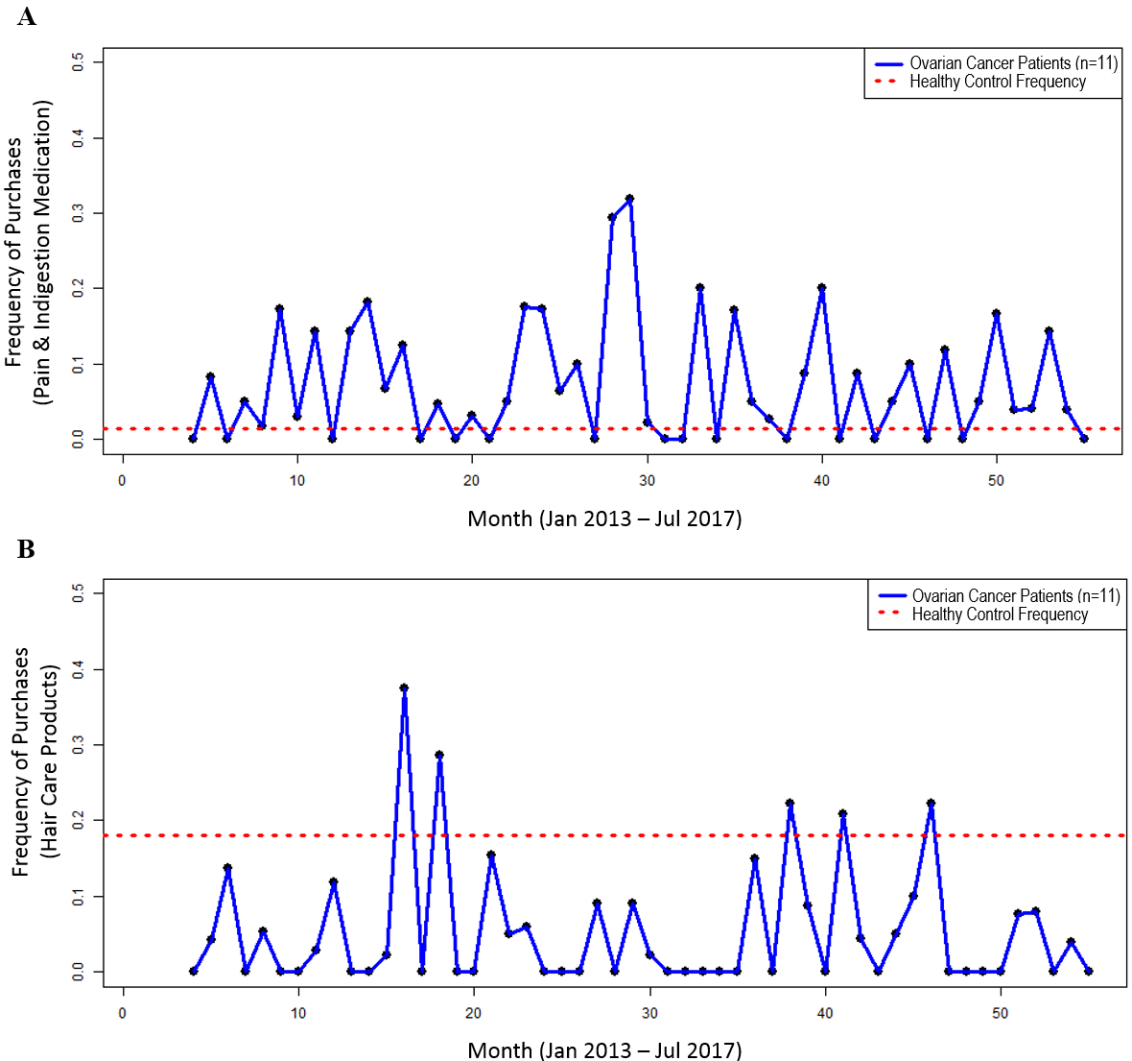
Overall purchase proportions. For each month between April 2013 and July 2017, the total purchases for each category were summed and divided by the number of all purchases in that month for the ovarian cancer patients (blue line), compared with the average monthly purchase proportion for that category for the healthy control subject (red dotted line). A. Purchases of pain and indigestion medication. B. Purchases of hair care products.

**Figure 2:**
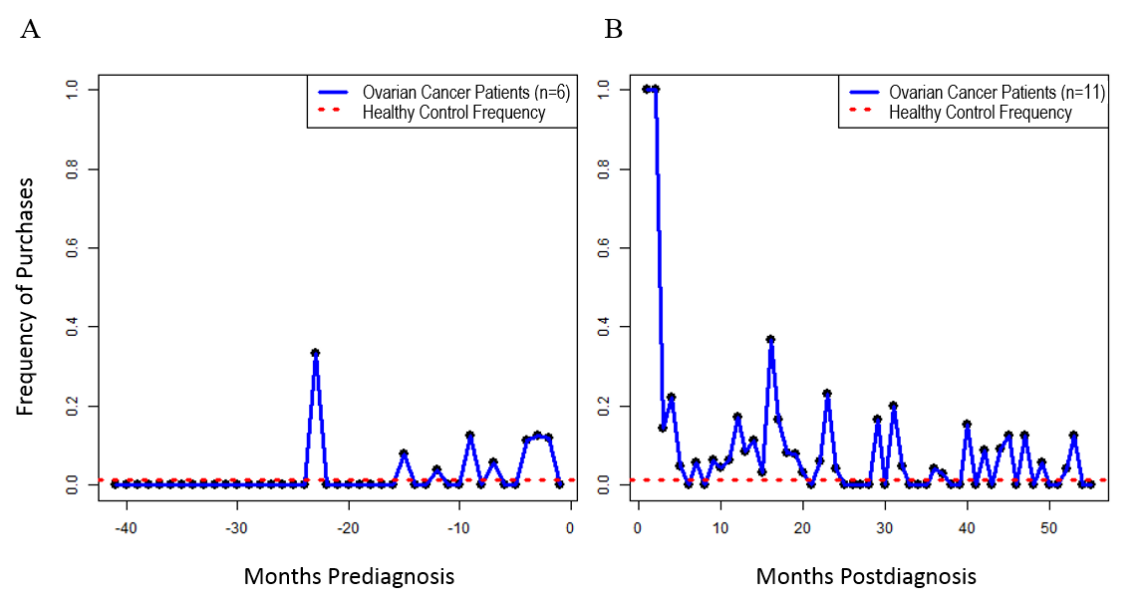
Pain and indigestion medication purchases stratified into pre- and postdiagnosis. Average monthly purchase proportions for ovarian cancer patients (blue line) were compared with those of the healthy control subject (red dotted line). A. Purchases for the pain and indigestion medication category during the prediagnostic period. B. Purchases for the pain and indigestion medication category during the postdiagnostic period.

Feasibility outcomes and participant characteristics were demonstrated using descriptive statistics. Due to variance in the frequency of purchases, we calculated the proportion of individual purchases matching the categories of interest—*hair care products* as one category and *pain plus indigestion medication* as the other category. For each category, the monthly ratio of each individual purchase to the overall purchases was computed. For example, the proportion of *pain plus indigestion medication* was calculated as *(pain medication + indigestion medication)/all purchases in the month*. The overall proportions, as reported in Figure 1, were calculated as averages for the calendar months across the study period. In Figure 2, patients were aligned with their diagnosis dates; an average proportion was calculated across the patients for each month prior to diagnosis (6/11, 55%) and postdiagnosis (11/11, 100%). Month and year of diagnosis were recorded for each ovarian cancer patient using the self-report survey and all purchase dates were aligned with pre- or postdiagnostic times. Where an ovarian cancer patient diagnosis was prior to the earliest purchase data, all data points were recorded as postdiagnosis from the date of the earliest purchase. The confidence interval of the mean was calculated using the R package *Publish* and ci.mean function. The data were analyzed using R version 3.2.3 (The R Foundation).

### Focus Group Study

#### Study Design and Setting

Between January and April 2017, we conducted four focus groups with members of the general public, 25-74 years of age, aiming to explore their acceptability of, and their attitudes toward, using commercial-data linkage for the purpose of cancer symptom surveillance. Participants from all parts of the United Kingdom were invited to attend a focus group based at the researchers’ offices at UCL in London, United Kingdom.

Research participants were recruited by placing an online recruitment advertisement on Twitter and Facebook, as well as by asking friends and colleagues of the researchers to share an email invitation. The online recruitment advertisement and email invitation promoted the fact that travel expenses would be reimbursed and focus groups would take place during lunchtime—with free refreshments included—so that participants could enjoy an afternoon in London afterward. Those interested in participating were instructed to click on a link to an online survey that asked individuals for their contact details and age. Individuals were selected for the study through the use of purposive sampling, which ensured that each focus group included individuals of different ages. Purposive sampling was used in this study, as previous research has shown a difference by age in the acceptability of sharing personal data, with younger individuals being more accepting of providing their personal information to commercial companies [25].

Each focus group was conducted for approximately one hour, facilitated by two members of the research team; all focus groups were led by one researcher, with another researcher assisting with facilitating the sessions. The role of the lead facilitator was to lead the discussion by asking the questions in the topic guide, encouraging all members to participate, and qualitatively balancing the amount of content that came from any one participant. The role of the assistant facilitator was to write field notes and to keep track of the timing.

At the beginning of each focus group session, participants were asked to complete a paper survey measuring demographic characteristics—age, gender, ethnicity, employment, and education—and use of the following: loyalty cards, online search engines, online shopping sites, health trackers, and social media (eg, Facebook, Twitter, and Instagram). The survey took no longer than five minutes to complete. The survey items have not been validated, but were included to allow us to describe the sample and to identify whether there were any biases in the groups (ie, if any group was overrepresented by participants of a particular demographic or by those who were more likely to use the apps or online services of interest).

Focus group questions were developed by the research team and reviewed for content and reliability. Two patient representatives from Cancer Research UK also reviewed the acceptability and the readability of the topic guide and provided further guidance. During the focus groups, the concept of data linkage was first introduced by asking participants how they felt about sharing their personal information with commercial companies and what they thought their data were used for. The discussion then moved on to asking participants about their attitudes toward researchers linking their commercial data with their health records to understand how their behaviors and that of others are linked to health conditions. The end of the discussion then focused more specifically on understanding participants’ thoughts on the potential to use commercial- and health-data linkage to predict cancer in the future using machine learning. The lead facilitator provided a description of this feasibility study in order for participants to understand the context for this discussion and the types of commercial data that may be used for the purpose of cancer surveillance in the future (eg, Fitbit and loyalty card data; see Multimedia Appendix 2).

#### Data Analysis

The focus groups were audiotaped and the audio files were transcribed verbatim. The researchers validated the accuracy of transcripts by comparing them with the audio files and the facilitators’ notes. The transcripts were analyzed using thematic analysis [26] using NVivo 11 software (QSR International). Interview transcripts were read repeatedly to extract themes, which were formatted into matrices to allow comparison of themes across participants and to identify the salient and prevalent dimensions of attitudes.

### Ethics Approval and Consent to Participate

Both studies have been reviewed by the University College London Research Ethics Committee and received favorable opinions (case study reference No. 6769/004 and focus group study reference No. 4657/002). The case study was also submitted to be reviewed by the CDRC Research Approvals Group (reference No. CDRC 018), which assessed the feasibility of the study and facilitated engagement with the high-street retailer. YH, JMF, and XS received the Safe User of Research Data Environments (SURE) training from the UK Data Service and had been subject to criminal records checks to receive permission to have access to data at the secure laboratories.

### Consent for Publication

We received individual consent from focus group participants to use their anonymized data in research publications, reports, webpages, and other research outputs. All anonymized outputs from the ovarian cancer case study were approved in accordance with CDRC data dissemination policies. Individual consent forms are being kept in a secure locker at YH’s department based at UCL for 10 years, in line with UCL’s data retention regulations.

## Results

### Ovarian Cancer Case Study

#### Feasibility Outcomes

Of the 70 patients who received the invitation, 18 women (26%) consented to take part in the study (see Table 1). Two people contacted the research team and reported not having a loyalty card as their reason for not participating. Of the 18 women who returned their consent, the median age was 55 (35-69) years and 17 women (94%) were white British. Of the 18 subjects, 7 (39%) had an unverified name or loyalty card number. We found that 17 out of the 18 (94%) participants recalled at least one symptom before their first visit to primary care; pain and fatigue were the most recognized symptoms. In total, purchase data from 11 ovarian cancer patients and one control subject were included in the final database. The high-street retailers retain individual purchase data for three years before aggregating the past purchase data. As a result, data from 1118 individual purchases were obtained from the retailer data ranging from April 2013 to July 2017. Of the final sample, 5 out of 11 patients (45%) were diagnosed before April 2013; therefore, all of their data were treated as postdiagnosis.

#### Proof-of-Concept Outcomes

Due to the higher patient recall of pain as one of the recognized symptoms before diagnosis, pain medication inclusive of indigestion and gastrointestinal tablets was chosen as the primary medication to monitor retrospectively. We selected *hair care products* as the control purchase category, which was expected to be unrelated to ovarian cancer symptoms. During the analysis period, there were 88 individual purchases of pain or indigestion medication. The monthly proportion of purchases of pain and indigestion medication in ovarian cancer patients ranged from 0% to 30% (8/27) across each of the months, in comparison to that of the healthy control subject, which accounted for approximately 1% (1/72) of all of their purchases (see Figure 1A). In comparison, there were 74 individual purchases of hair care products among the purchases. Hair care products accounted for approximately 18% (13/72) of all purchases by the healthy control compared with 0% to 38% (9/24) each month for the ovarian cancer patients (see Figure 1B).

To test for self-care behaviors before diagnosis, we split the timeline and demonstrated the purchasing trends by calculating the purchases for each month pre- and postdiagnosis. Figure 2 shows that around 12 months before diagnosis, women started purchasing pain and indigestion medication, while their behavior is the same as the healthy control individual before their potential nonapparent symptoms might have started to present themselves. We found pain and indigestion medication representing 12 out of 202 (5.9%, 95% CI 1.0-8.8) purchases prediagnosis and 73 out 1011 (7.22%, 95% CI 4.5-15.0) purchases postdiagnosis, compared with the healthy control at 1 out of 72 (1%) purchases (see Figure 2). In comparison, the hair care products represented 24 out of 202 (11.9%, 95% CI 5.3-26.7) purchases before diagnosis and 37 out of 1011 (3.66%, 95% CI 2.3-6.6) purchases postdiagnosis in the ovarian cancer patients, compared with 13 out of 72 (18%) purchases in the healthy control.

### Focus Group Studies

#### Acceptability of Commercial- and Health-Data Linkage for Cancer Symptom Surveillance

In total, 27 people took part in one of four focus groups (see Table 2). Overall, 19 out of the 27 participants (70%) were female with at least one to three male participants in each group. Distribution of participant characteristics is presented in Table 2. Four key themes were identified from the discussions in all the focus groups: conditional acceptance of commercial- and health-data linkage and symptom surveillance, beliefs about accuracy of the data, perceived benefits, and considerations for communication strategies.

**Table 1:** Ovarian cancer case study participant characteristics.

Participant characteristics		All respondents (N=18)	Ovarian cancer patients (N=11)
Age in years, median (range)		55 (35-69)	56 (35-69)
**Ovarian cancer diagnosis, n (%)**			
	Yes	17 (94)	11 (100)
	No	1 (6)	0 (0)
**Ethnicity, n (%)**			
	White	17 (94)	10 (91)
	Missing	1 (6)	1 (9)
**Symptoms observed before diagnosis (all respondents, N=17), n (%)**			
	Any	15 (88)	9 (82)
	Irregular periods	1 (6)	1 (9)
	Pain (back, tummy, urinary, during sex)	12 (71)	6 (55)
	Constipation	1 (6)	1 (9)
	Weight loss	1 (6)	1 (9)
	Bloating	3 (18)	1 (9)
	Loss of appetite	4 (24)	2 (18)
	Fatigue	7 (41)	6 (55)
**Perceived health, n (%)**			
	Excellent to very good	4 (22)	3 (27)
	Good to fair	10 (56)	5 (45)
	Poor	4 (22)	4 (36)
**Loyalty card use, n (%)**			
	All the time/often	13 (72)	8 (72)
	Sometimes/not very often	4 (22)	2 (18)
	Not at all	1 (6)	1 (9)
**Subscription to loyalty cards provided by high-street retailers, n (%)**			
	Tesco	17 (94)	10 (91)
	Boots	15 (83)	11 (100)
	Sainsbury	13 (72)	8 (73)
	All three above	10 (56)	7 (64)
	Coop	8 (44)	4 (36)
	Morrison	1 (6)	1 (9)
	Superdrug	4 (22)	3 (27)

**Table 2:** Focus group study participant characteristics.

Participant characteristics		Participants (N=27), n (%)
**Focus group distribution**		
	Group 1	5 (19)
	Group 2	6 (22)
	Group 3	7 (26)
	Group 4	9 (33)
Age in years, median (range)		55 (25-71)
**Gender **		
	Male	8 (27)
	Female	19 (70)
**Ethnicity**		
	White British	25 (93)
	Other white	1 (4)
	Other mixed	1 (4)
**Employment**		
	Full-time employee	13 (48)
	Part-time employee	3 (11)
	Retired	7 (26)
	Student	2 (7)
	Disabled or too ill to work	1 (4)
	Full-time homemaker	1 (4)
**Education**		
	GCSE^a^/O Level^b^/CSE^c^, vocation qualifications, or A Level^d^	6 (22)
	Higher education (degree or higher)	18 (67)
	No formal qualifications	3 (11)
**Number of loyalty cards**		
	None	10 (37)
	One card	2 (7)
	Between two and five cards	11 (41)
	More than five cards	4 (15)
**Use of Facebook**		
	Yes	17 (63)
	No	10 (37)
**Use of Instagram**		
	Yes	7 (26)
	No	20 (74)
**Use of online search engines**		
	Yes	25 (93)
	No	2 (7)
**Use of online shopping**		
	Yes	19 (70)
	No	8 (30)
**Use of health trackers**		
	Yes	10 (37)
	No	17 (63)
**Use of Twitter**		
	Yes	10 (37)
	No	17 (63)

^a^GCSE: General Certificate of Secondary Education.

^b^O Level: Ordinary Level.

^c^CSE: Certificate of Secondary Education.

^d^A Level: Advanced Level.

#### Conditional Acceptance of Commercial- and Health-Data Linkage and Symptom Surveillance

In general, the concept of linking commercial and health data for early detection of cancer was perceived to be interesting and people were conditionally willing to share their data. The key conditions were having safeguards, transparency, and an option to opt out or withdraw from the study. Safeguards included not just data security, but also ensuring that the data would not be used for purposes outside of the details provided in the initial consent, as illustrated in the following quote:

If somebody checked the data on my phone, because we had a cancer scare, it would be quite interesting because I had gone on symptom checkers quite a lot, so I think they’d have got quite a lot of data probably from that, which might have been useful. So, I wouldn’t disagree with that, it’s always this thing of safeguards isn’t it?

Focus Group 4, Participant 27, female, age 66

The conditions also included transparency about the data management and data sharing policies. Participants wanted clear and concise information about the purpose and usage of their data during the consent process. These were all related to concerns about the misuse of the data by commercial gain.

I wouldn’t be averse to that for research purposes, people being able to link things, as long as only certain people had access to that and that it was well-controlled and [with] data protection.

Focus Group 2, Participant 10, female, age 34

They do loads of these terms and conditions because they know you’ll get bored before the end of it or they’ll confuse you before you get to the end of it. They should make that very clear at the box that it’s only for their [researchers’] use.

Focus Group 3, Participant 12, female, age 55

#### Beliefs About Accuracy of the Data

While participants were intrigued by the potential to detect cancer early through data linkages between commercial datasets and health records, there was skepticism about the accuracy of the data as well as the potential outcomes of misinterpretation. This concern was toward the predictive utility of understanding illness development, the presence of symptoms, and behavior change, using data other than individual health records.

Do we understand yet a cure for cancer as a result of some really good researchers and all our data? I think that kind of story would be really convincing.

Focus Group 3, Participant 17, male, age 53

Relating to the predictive utility, participants questioned whether the commercial data will be representative of the individuals’ actions and the symptoms they could be experiencing. The reliability of data entered into the social media websites and search engines were questioned by most focus groups. In particular, concerns were raised about data entry on behalf of someone else or for interest (eg, looking up someone else’s symptoms on search engines). Participants also felt that people are not open and honest about their actual behaviors on social media websites and agreed that outcomes of social media data analysis will have a “self-presentation bias.” In most focus groups, participants proposed a preference and trust in objective data (eg, tracker data and phone apps).

People start having the symptoms and they change their eating habits, get more yoghurts or cut down on the bread and things; could that not just be that our taste buds change and we like bread for a while and then go, “I’m fed up of bread.” And there’s actually nothing wrong with us, it’s just...

Focus Group 3, Participant 12, female, age 55

#### Perceived Benefits

Participants agreed that if cancer symptom surveillance is found to be effective, there may be a positive impact on research, an increase in early diagnosis, and ultimately reduction in costs to the National Health Service. A few mentioned that this could reduce the pressure on emergency services and could support general practitioners’ (GPs) decision-making processes if they had a clear idea of the symptoms timeline. One participant, however, perceived the impact to be more direct on his life and expressed the potential impact that this research would have on his partner and himself if her online data and consumer behavior were researched before she was diagnosed with cancer.

So she started to feel tired, so she’s Googling tiredness, but privately; then she’s got this pain in her shoulders, so she started having physio on her shoulders—this is from September to February. She’s buying some painkillers or whatever and eventually a lump appears under her arm and she went to the doctor and it’s late-stage lung cancer; but she’s a very fit woman, so in those six months beforehand, you look back now in time on reflection, it’s pretty obvious that pain she was having was the tumor. So that makes sense doesn’t it? It might give her a heads up, “you’ve got a tumor here,” or “get checked out for a tumor.”...So that early diagnosis makes complete sense, however, is it for us as human beings to discover stuff? I don’t know. I’m really interested to hear.

Focus Group 3, Participant 17, male, age 53

#### Considerations for Communication Strategies

Furthermore, the discussions included how people would like to be informed about the outcomes if such analysis existed in the future. Some participants preferred being directly informed from a trusted source (eg, their GP). They felt direct letters with a GP’s recommendation to themselves would prompt an action toward early detection or prevention. Others preferred to be informed by receiving a generalized public health message where the outcome could be more informative rather than used to highlight specific risk.

If someone found out something might be a pointer towards a problem, I’d like it to go through the proper channels and come from my GP rather than anyone else really.

Focus Group 4, Participant 23, male, age 60

I like to think if it was a very good advert, maybe some compelling way of communicating with people, then I would. I also feel like doing something in a community that feels nicer to me than getting a horrible email.

Focus Group 3, Participant 16, female, age 26

All participants felt that feedback from either of the options would have to be communicated clearly to ensure that it does not create any unwarranted anxiety among those who are not actually having symptoms of cancer.

I’m just thinking that it might be too vague and that you might give people an idea that they could have cancer who are actually not at risk at all. I’m just thinking that it might actually cause more anxiety in people than it would do good.

Focus Group 2, Participant 8, female, age 25

## Discussion

### Principal Findings

This study demonstrates the potential to investigate patient appraisal before someone starts having any symptoms and signs related to cancer using real-time data collected by commercial organizations. Our study showed that real-time data collected by a commercial organization could offer insights to patients before presentation at primary care. Furthermore, if this data are used fairly and if the processes are transparent, the public are willing to give consent to commercial- and health-data linkages. It is also important to note that although it is feasible to investigate commercial- and health-data linkages, there needs to be further developments toward public trust in data accuracy and communication strategies.

As stated, screening for ovarian cancer is not being recommended [10] and the early detection of ovarian cancer still remains a major public health problem. Although our study had a limited sample size to detect differences between the cases and the control group, we did observe purchases of pain and indigestion medication in the ovarian cancer patients leading up to diagnosis. Our findings are encouraging to pursue the monitoring of self-care behaviors of ovarian cancer patients with a large-scale, retrospective, case-control study. Although the focus groups agreed that this data linkage was acceptable, only 26% of the ovarian cancer patients approached for this study consented to participate. One of the reasons for not consenting may have been that they did not hold any requisite loyalty cards, but this will need to be explored in future research. We believe that past literature on self-care behaviors before diagnosis and the emerging evidence supports this research agenda. For instance, a recent study on the nature and the frequency of abdominal symptoms suggest that patients with persistent bloating and distention waited a minimum of two months before presenting to primary care [27]. The identification of self-care behaviors using commercial data could be an effective approach to probe earlier engagement in primary care. For ovarian cancer patients, specifically, this might mean an increase in purchase of antacids to alleviate the feeling of indigestion associated with bloating symptoms. It might also mean a prolonged chronic use of pain medication to alleviate stomach pain or back pain associated with ovarian cancer.

Furthermore, access to real-life data through high-street retailers, trackers, and mobile phone apps will also open up other opportunities for future research. For example, the link between diet and cancer risk has been extensively studied in epidemiological cohort studies, such as the European Prospective Investigation into Cancer and Nutrition [28]. These studies have traditionally used food frequency questionnaires to estimate links between individuals’ diets and cancer incidence, which have considerable recall bias and often only measure at very few time points. Many other cancer types also have specific symptoms that might be alleviated by over-the-counter medications or monitored using loyalty card data. For example, symptomatic esophageal cancer is often mistaken for indigestion and gastroesophageal reflux [29], lung cancer is often mistaken for persistent coughing [30], and pancreatic cancer is often mistaken for abdominal pain and loss of appetite [31]. Furthermore, with a large enough cohort using an agnostic approach with machine learning, one could discover novel purchase behaviors associated with early cancer symptoms.

### Strengths and Limitations

This proof-of-concept study was the first-ever research project that aimed to understand self-care behaviors of cancer patients prior to their diagnosis using commercial data. Therefore, we have learned about the limitations of our proposed methodology as we proceeded with the data collection. The limitations of our study includes the small number of subjects that were available for analysis of loyalty card data. Our data does not show evidence of distinguishing between ovarian cancer patients and control subjects given the small number of subjects. However, it does show that it is feasible to analyze loyalty card data for purchases such as pain and indigestion medication by patients prior to their diagnoses. Furthermore, it is also important to clarify that identifying these purchases are not sufficient to diagnose ovarian cancer, but should be sufficient to *nudge* the patient to visit their GPs and discuss these symptoms as a potential cancer-related symptom. Larger studies will be needed to assess any statistical evidence to support our hypothesis that purchase behavior may indicate cancer symptoms prior to diagnosis and to assess the sensitivity and specificity of detecting a cancer diagnosis. In retrospect, hair care products were not an ideal comparator, particularly for the postdiagnosis period, as there is a period during chemotherapy when hair care is not particularly relevant to ovarian cancer patients, although it is still relevant for the prediagnostic period. Other product categories may be needed as control purchases for future studies. Lastly, by using individual consent to analyze purchase behaviors, we have also identified the most secure pathway to analyze commercial data, which also fulfilled the criteria for the commercial organization and the participants.

As the participants recruited for the focus groups were self-selected, this may have introduced bias into the sample. Although the focus groups were relatively diverse, with a broad age range and a mixture of socioeconomic groups, the sample was unbalanced for gender (70% female). This gender imbalance is also observed with loyalty card usage, with the majority of card holders from most high-street retailers being female, which offers an insight about the target population for using loyalty card data. The use of loyalty cards as a data source, in general, has other limitations that need to be explored further. These include the fact that people often buy for other family members, not just themselves; they do not always use the card for every purchase; they may often shop at other stores; or they may not even hold any loyalty cards. Based on our data, approximately half of the women held multiple loyalty cards from several retailers and for these individuals the use of loyalty card data will be of most value when combining data from several sources. When conducting future studies, we will require the collaboration of data analysts at multiple commercial organizations to understand the variation in household data (eg, the proportion of individuals who buy products on behalf of others and a way to combine loyalty card data from multiple retailers to understand an individuals’ purchasing behavior more clearly). With the new General Data Protection Regulation by the European Union and support by our focus group outcomes on transparency and accountability, any other use of loyalty card data and data linkage needs to be conducted with individual consent and in a secure environment. Although this may be perceived as a barrier to conducting large-scale projects or big data analyses, we were able to fulfill focus group participants’ and supporting retailers’ criteria with our proposed methodology using the CDRC secure laboratory.

### Conclusions

In summary, we have shown that the potential use of commercial- and health-data linkage for cancer symptom surveillance was generally acceptable, with assurances for transparency, security, and confidentiality. Our use of individual purchase data, from loyalty card data from a high-street retailer, was an appropriate source of this data to explore this novel method for earlier diagnosis of ovarian cancer. There are a number of exciting opportunities to use this data to investigate novel methods of cancer surveillance and symptom recognition. For example, unbiased machine learning-based approaches may be used to discover novel purchase behaviors or interactions between variables in these datasets to develop new hypotheses that can be tested. Lastly, understanding when ovarian cancer patients begin to self-medicate symptoms may provide more direct empirical evidence for when symptoms occur prior to diagnosis and improve our understanding of the natural progression of this disease.

### Availability of the Data and Material

The case study data for this research have been provided by the CDRC, an ESRC Data Investment, under project ID CDRC 0018, ES/L011840/1; ES/L011891/1. Under CDRC license agreement, the data included in the ovarian cancer case study was limited to the purposes of this project and cannot be shared with others. The anonymized focus group transcripts can be made available from the corresponding author upon reasonable request and will be assessed on a case-by-case basis. Users will be required to complete a data-sharing agreement.

## Appendix

Multimedia Appendix 1 Patient pathway recruitment survey.

Multimedia Appendix 2 Focus group discussion guide.

## Abbreviations

A Level: Advanced Level
CA 125: cancer antigen 125
CDRC: Consumer Data Research Centre
CSE: Certificate of Secondary Education
ESRC: Economic and Social Research Centre
GCSE: General Certificate of Secondary Education
GP: general practitioner
O Level: Ordinary Level
SURE: Safe User of Research Data Environments
UCL: University College London

## References

[1] Whitaker KL, Macleod U, Winstanley K, Scott SE, Wardle J (2015). Help-seeking for cancer "alarm" symptoms: A qualitative interview study of primary care patients in the UK. Br J Gen Pract.

[2] Whitaker KL, Winstanley K, Macleod U, Scott SE, Wardle J (2015). Low cancer suspicion following experience of a cancer "warning sign". Eur J Cancer.

[3] Winstanley K, Renzi C, Smith CF, Wardle J, Whitaker KL (2016). The impact of body vigilance on help-seeking for cancer "alarm" symptoms: A community-based survey. BMC Public Health.

[4] Renzi C, Whitaker KL, Wardle J (2015). Over-reassurance and undersupport after a "false alarm": A systematic review of the impact on subsequent cancer symptom attribution and help-seeking. BMJ Open.

[5] Ebell MH, Culp MB, Radke TJ (2016). A systematic review of symptoms for the diagnosis of ovarian cancer. Am J Prev Med.

[6] Goff B (2012). Symptoms associated with ovarian cancer. Clin Obstet Gynecol.

[7] Low EL, Whitaker KL, Simon AE, Sekhon M, Waller J (2015). Women's interpretation of and responses to potential gynaecological cancer symptoms: A qualitative interview study. BMJ Open.

[8] (2016). Cancer Research UK.

[9] Buys SS, Partridge E, Black A, Johnson CC, Lamerato L, Isaacs C, Reding DJ, Greenlee RT, Yokochi LA, Kessel B, Crawford ED, Church TR, Andriole GL, Weissfeld JL, Fouad MN, Chia D, O'Brien B, Ragard LR, Clapp JD, Rathmell JM, Riley TL, Hartge P, Pinsky PF, Zhu CS, Izmirlian G, Kramer BS, Miller AB, Xu J, Prorok PC, Gohagan JK, Berg CD, PLCO Project Team (2011). Effect of screening on ovarian cancer mortality: The Prostate, Lung, Colorectal and Ovarian (PLCO) Cancer Screening Randomized Controlled Trial. JAMA.

[10] Jacobs IJ, Menon U, Ryan A, Gentry-Maharaj A, Burnell M, Kalsi JK, Amso NN, Apostolidou S, Benjamin E, Cruickshank D, Crump DN, Davies SK, Dawnay A, Dobbs S, Fletcher G, Ford J, Godfrey K, Gunu R, Habib M, Hallett R, Herod J, Jenkins H, Karpinskyj C, Leeson S, Lewis SJ, Liston WR, Lopes A, Mould T, Murdoch J, Oram D, Rabideau DJ, Reynolds K, Scott I, Seif MW, Sharma A, Singh N, Taylor J, Warburton F, Widschwendter M, Williamson K, Woolas R, Fallowfield L, McGuire AJ, Campbell S, Parmar M, Skates SJ (2016). Ovarian cancer screening and mortality in the UK Collaborative Trial of Ovarian Cancer Screening (UKCTOCS): A randomised controlled trial. Lancet.

[11] Hamilton W, Peters TJ, Bankhead C, Sharp D (2009). Risk of ovarian cancer in women with symptoms in primary care: Population-based case-control study. BMJ.

[12] Goff BA, Mandel L, Muntz HG, Melancon CH (2000). Ovarian carcinoma diagnosis. Cancer.

[13] Brandner S, Müller-Nordhorn J, Stritter W, Fotopoulou C, Sehouli J, Holmberg C (2014). Symptomization and triggering processes: Ovarian cancer patients' narratives on pre-diagnostic sensation experiences and the initiation of healthcare seeking. Soc Sci Med.

[14] Walter F, Webster A, Scott S, Emery J (2012). The Andersen Model of Total Patient Delay: A systematic review of its application in cancer diagnosis. J Health Serv Res Policy.

[15] Atienza AA, Serrano KJ, Riley WT, Moser RP, Klein WM (2016). Advancing cancer prevention and behavior theory in the era of big data. J Cancer Prev.

[16] Eysenbach G (2009). Infodemiology and infoveillance: Framework for an emerging set of public health informatics methods to analyze search, communication and publication behavior on the Internet. J Med Internet Res.

[17] Rice RE (2006). Influences, usage, and outcomes of Internet health information-searching: Multivariate results from the Pew surveys. Int J Med Inform.

[18] Ginsberg J, Mohebbi MH, Patel RS, Brammer L, Smolinski MS, Brilliant L (2009). Detecting influenza epidemics using search engine query data. Nature.

[19] Chan EH, Sahai V, Conrad C, Brownstein JS (2011). Using Web search query data to monitor dengue epidemics: A new model for neglected tropical disease surveillance. PLoS Negl Trop Dis.

[20] White RW, Tatonetti NP, Shah NH, Altman RB, Horvitz E (2013). Web-scale pharmacovigilance: Listening to signals from the crowd. J Am Med Inform Assoc.

[21] Liu S, Hodgson C, Zbib AM, Payne AYM, Nolan RP (2014). The effectiveness of loyalty rewards to promote the use of an Internet-based heart health program. J Med Internet Res.

[22] Paul MJ, White RW, Horvitz E (2014). Search and Breast Cancer: On Disruptive Shifts of Attention Over Life Histories of an Illness.

[23] Paparrizos J, White RW, Horvitz E (2016). Screening for pancreatic adenocarcinoma using signals from Web search logs: Feasibility study and results. J Oncol Pract.

[24] Schmidt B (2006). Proof-of-principle studies. Epilepsy Res.

[25] Castell S, Charlton A, Clemence M, Pettigrew N, Pope S, Quigley A, Shah JN, Silman T (2014). Public Attitudes to Science 2014.

[26] Braun V, Clarke V (2006). Using thematic analysis in psychology. Qual Res Psychol.

[27] Koo MM, von Wagner C, Abel GA, McPhail S, Hamilton W, Rubin GP, Lyratzopoulos G (2018). The nature and frequency of abdominal symptoms in cancer patients and their associations with time to help-seeking: Evidence from a national audit of cancer diagnosis. J Public Health (Oxf).

[28] Gonzalez CA, Riboli E (2010). Diet and cancer prevention: Contributions from the European Prospective Investigation into Cancer and Nutrition (EPIC) study. Eur J Cancer.

[29] Lagergren J, Bergström R, Lindgren A, Nyrén O (1999). Symptomatic gastroesophageal reflux as a risk factor for esophageal adenocarcinoma. N Engl J Med.

[30] Corner J, Hopkinson J, Fitzsimmons D, Barclay S, Muers M (2005). Is late diagnosis of lung cancer inevitable? Interview study of patients' recollections of symptoms before diagnosis. Thorax.

[31] Gullo L, Tomassetti P, Migliori M, Casadei R, Marrano D (2001). Do early symptoms of pancreatic cancer exist that can allow an earlier diagnosis?. Pancreas.

