# Supplementary material for: Self-Care Behaviors of Ovarian Cancer Patients Before Their Diagnosis: Proof-of-Concept Study
Source: JMIR Cancer. 2019 Jan 17;5(1):e10447. doi: 10.2196/10447 (PMC6354198; doi:10.2196/10447)
Supplement: Multimedia Appendix 1 [file cancer_v5i1e10447_app1.pdf]

Participant number:

CRUK Guardian Angel Participant Questionnaire

Thank you for agreeing to complete this survey.

- It should take no longer than 10 minutes to complete. You may, however, take as long as you wish.
- There are no right or wrong answers and it does not matter whether you change your responses.
- Your answers will be sent directly to the UCL research team and will be treated in strict confidence.
- Please return the survey in the freepost envelope (no stamp required).

If you have any difficulties completing this survey or have any questions about it, please call **020 7679 1615 or send an email to the principal researcher, Yasemin Hirst, y.hirst@ucl.ac.uk**

Thank you

The first few questions is about your experience with symptoms and bodily changes prior to your cancer diagnosis

As mentioned in our invitation sheet, in order to evaluate whether or not there were changes during the first time you thought you are experiencing changes in your body and until you first made your first appointment with your GP we need to put together an individual timeline for each patient we invited to this project.

First time I felt changes in my body was approximately in

- Month:
- Year :

I made an appointment with my doctor about these symptoms and changes in

- Month :
- Year :

I received my diagnosis in

- Month :
- Year:

**Was there a particular symptom or symptoms that you felt when you go back to <u>the first time</u> you felt changes in your body (i.e. way before you went to your GP)?"**	
☐ YES	☐ NO

If YES, please indicate the first symptom or symptoms you recognised prior to your visit to your GP

☐ Chest pain/ Persistent Cough/Sore throat/
☐ Shortness of breath
☐ Unexplained Lump
☐ Unexplained bleeding
☐ Persistent unexplained pain
☐Back Pain
☐Lower tummy (abdominal) pain
☐Headache
☐ Change in the appearance of a mole
☐ Difficulty swallowing
☐Passing urine more than usual
☐Constipation
☐Pain during sex
☐ Weight loss
☐ Irregular Periods or vaginal bleeding after menopause (women only)
☐A swollen abdomen / Persistent bloating
☐Feeling of fullness or loss of appetite
☐Feeling tired/constant fatigue
☐Persistent change in Bowel Habits
☐Other_______________________________
☐ Other_______________________________

Have you used any over-the-counter medication to manage the symptom or symptoms without seeing your GP?

☐ **Yes**
Please Specify : _____________________________

☐ **No, I did not use medication**
☐ **Waited for the symptom to resolve on it is own**

☐ **I used other methods**
Please Specify : _____________________________

☐ **I used non-medicinal (natural) remedies (e.g.**
Specify : _____________________________

☐ **Other**
Specify : _____________________________

FINALLY, A FEW QUESTIONS ABOUT YOU TO HELP US ANALYSE THE RESULTS OF THE SURVEY

Please enter today's date: d d / m m / y y y y

What is your age? ☐☐

Which of these best describes your ethnic group?

White		Mixed		Asian or Asian British		Black or Black British		Chinese/ other	
☐	White British	☐	White and Black Caribbean	☐	Indian	☐	Black Caribbean	☐	**Chinese**
☐	White Irish	☐	White and Black African	☐	Pakistani	☐	Black African	☐	**Other………**
☐	Any other White background	☐	White and Asian	☐	Bangladeshi	☐	Any other Black background	☐	**Prefer not to say**
		☐	**Any other Mixed background**	☐	**Any other Asian background**				

What is your marital status?

Single/ never married	Married/ living with partner	Divorced/ Seperated	Widowed	Prefer not to say
☐	☐	☐	☐	☐

What is the highest level of education qualification you have obtained?			
☐	Degree or higher degree	☐	**O Level or GCSE equivalent (Grade A - C)**
☐	Higher education qualification below degree level	☐	**O Level or GCSE (Grade D - G)**
☐	A-levels or highers	☐	**No formal qualifications**
☐	ONC/BTEC	☐	**Other ……………………….**
☐	**Still studying**	☐	**Prefer not to say**

Have you, your family or close friends had cancer?				
	Yes	No	Don't know	**Prefer not to say**
**You**	☐	☐	☐	☐
**Partner**	☐	☐	☐	☐
**Close family member**	☐	☐	☐	☐
**Other family member**	☐	☐	☐	☐
**Close friend**	☐	☐	☐	☐
**Other friend**	☐	☐	☐	☐

Compared with other people your age, would you say your health is …				
Poor	Fair	Good	Very good	Excellent
☐	☐	☐	☐	☐

Do you have a different long standing/Chronic illness which requires constant medication?	
Yes ☐	No ☐
Please tell us what it is	

How often do you use loyalty cards when you go shopping?				
Not at all ☐	Not very often ☐	Sometimes ☐	Often ☐	All the time ☐

Which of the following cards do you own?

☐ Tesco
☐ Boots
☐ Sainsbury’s (Nectar Card)
☐ Co-op
☐ Morrison’s
☐Superdrug
☐ Other ___________________
☐ Other ___________________
☐ Other ___________________
☐ Other ___________________
☐ Other ___________________
☐ Other ___________________

Thank you for completing this survey.
