# Supplementary material for: Self-Care Behaviors of Ovarian Cancer Patients Before Their Diagnosis: Proof-of-Concept Study
Source: JMIR Cancer. 2019 Jan 17;5(1):e10447. doi: 10.2196/10447 (PMC6354198; doi:10.2196/10447)
Supplement: Multimedia Appendix 2 [file cancer_v5i1e10447_app2.pdf]

“Can we use your commercial data to monitor possible cancer symptoms?”

Discussion guide for a focus group study

Overview of study and ground rules

Hello, my name is [researcher’s name] and this is [researcher’s name], and we work at the Health Behaviour Research Centre at University College London. The purpose of this focus group is to explore your views on providing personal information to commercial organisations (e.g. Tesco’s, Amazon, Facebook – in other words organisations that provide a service for profit), and how you feel about the ways this information is used by organisations. We would like to encourage all of you to go into as much detail as you can, what you have to say is very valuable information.

The focus group session will last approximately one hour. Everything you say is strictly confidential and anonymous. There are no right or wrong answers; I simply want to hear your views. I am not here to judge your comments but rather to collect them. Quotes may be used when the results of the study are being presented or published, but again you will not be personally identified.

Are you happy for me to record this interview?

If No, will take notes (or terminate interview if notes are not allowed)

If Yes…..

PRESS RECORD

Prompts: advantages/disadvantages, type of data, trust, how they think data is being used, concerns, willingness to consent,

1) How do you feel about providing your personal data to commercial organisations?

2) *In the distant or possibly the near future, researchers may want to link your commercial data with your health records to understand how your behaviours as well as others are link to health conditions. This may include linking your health records with location data collected by your mobile GPS data, purchasing data collected when using your loyalty cards, data from search engine logs collected when typing search terms in websites such as google.*

What are your immediate thoughts and feelings about this data linkage?

3) *Let’s say there is a mobile phone app that you can use to consent researchers to link your health records (clinical) with your Fitbit data (pedometers) and your purchasing data using your loyalty card just by ticking a box. It will have a tick box per dataset and relevant information about how your data are being used. You can revise your consent, add or remove datasets as you may wish.*

How would you feel about providing your informed consent for data linkage through a mobile phone application?

4) *Myself and my colleagues are conducting a feasibility study that aims to investigate whether or not cancer can be predicted using commercial data. We want to investigate whether the changes in purchases (e.g. increased purchases of medication), online searches, social media submissions, and pictures etc. can predict whether someone is experiencing symptoms of cancer or another illness that could be serious. Ultimately we want to inform individuals to recognise that the bodily changes they are experiencing could be a sign of cancer at the earliest opportunity possible. Bodily changes could include: sensation, weight loss or gain, cough, unexplained pain and/or bloating. However there is no concrete evidence of how individuals self-manage their bodily changes before they decide it is time to seek help from a health professional. We aim to use existing commercial data to understand self-management behaviours further.*

What are your immediate thoughts about this study?

End of focus group – Stop recording
